# Transplantation of Human Umbilical Mesenchymal Stem Cells from Wharton's Jelly after Complete Transection of the Rat Spinal Cord

**DOI:** 10.1371/journal.pone.0003336

**Published:** 2008-10-06

**Authors:** Chang-Ching Yang, Yang-Hsin Shih, Miau-Hwa Ko, Shao-Yun Hsu, Henrich Cheng, Yu-Show Fu

**Affiliations:** 1 Institute of Anatomy and Cell Biology, School of Medicine, National Yang-Ming University, Taipei, Taiwan, Republic of China; 2 Department of Neurosurgery, Neurological Institute, Taipei Veterans General Hospital, Taipei, Taiwan, Republic of China; 3 School of Medicine, Taipei Medical University, Taipei, Taiwan, Republic of China; 4 Department of Anatomy, School of Medicine, China Medical University, Taichung, Taiwan, Republic of China; 5 Neural Regeneration Laboratory, Department of Neurosurgery, Neurological Institute, Taipei Veterans General Hospital, Taiwan, Republic of China; 6 Department of Pharmacology, School of Medicine, National Yang-Ming University, Taipei, Taiwan, Republic of China; 7 Department of Anatomy and Cell Biology, School of Medicine, National Yang-Ming University, Taipei, Taiwan, Republic of China; 8 Department of Education and Research, Taipei City Hospital, Taipei, Taiwan, Republic of China; Copenhagen University Hospital, Denmark

## Abstract

**Background:**

Human umbilical mesenchymal stem cells (HUMSCs) isolated from Wharton's jelly of the umbilical cord can be easily obtained and processed compared with embryonic or bone marrow stem cells. These cells may be a valuable source in the repair of spinal cord injury.

**Methodology/Principal Findings:**

We examine the effects of HUMSC transplantation after complete spinal cord transection in rats. Approximately 5×10^5^ HUMSCs were transplanted into the lesion site. Three groups of rats were implanted with either untreated HUMSCs (referred to as the stem cell group), or HUMSCs treated with neuronal conditioned medium (NCM) for either three days or six days (referred to as NCM-3 and NCM-6 days, respectively). The control group received no HUMSCs in the transected spinal cord. Three weeks after transplantation, significant improvements in locomotion were observed in all the three groups receiving HUMSCs (stem cell, NCM-3 and NCM-6 days groups). This recovery was accompanied by increased numbers of regenerated axons in the corticospinal tract and neurofilament-positive fibers around the lesion site. There were fewer microglia and reactive astrocytes in both the rostral and caudal stumps of the spinal cord in the stem cell group than in the control group. Transplanted HUMSCs survived for 16 weeks and produced large amounts of human neutrophil-activating protein-2, neurotrophin-3, basic fibroblast growth factor, glucocorticoid induced tumor necrosis factor receptor, and vascular endothelial growth factor receptor 3 in the host spinal cord, which may help spinal cord repair.

**Conclusions/Significance:**

Transplantation of HUMSCs is beneficial to wound healing after spinal cord injury in rats.

## Introduction

Mammalian spinal cord injury is followed by the degeneration of axons, loss of neurons and glia, and demyelination around the lesion site. Axonal regeneration in the central nervous system (CNS) is impeded partly by myelin-associated inhibitors [1]–[2] and formation of a post-lesion scar barrier [3]. The extent of intrinsic cell renewal alone [4], even after application of mitogenic agents such as epidermal growth factor and fibroblast growth factor-2 [5], [6], is not sufficient to allow substantial recovery following spinal cord injury [7]. Therefore, therapeutic strategies that involve exogenous cell replacement have to be considered.

Mesenchymal cells from Wharton's jelly of the umbilical cord possess stem cell properties [8]–[10]. We previously demonstrated that human umbilical mesenchymal stem cells (HUMSCs) could be induced to differentiate into neuron-like cells (about 87%), express neurofilament and functional mRNAs responsible for the syntheses of subunits of the kainate receptor and glutamate decarboxylase, and generate an inward current in response to evocation by glutamate [9]. HUMSCs are also capable of differentiating into osteogenic, chondrogenic, adipogenic, and myogenic cells *in vitro* [10]. We also found that the transformed HUMSCs in the striatum were still viable 4 months after transplantation without the need for immunological suppression, suggesting that HUMSCs might be a good stem cell source for transplantation [11].

In this study, we evaluate the effect of transplantation of HUMSCs on axon regeneration in the injured spinal cord using a complete transection model in rats. Cultures of HUMSCs were treated with neuronal conditioned medium (NCM) for 0, 3 or 6 days, and then were implanted to the lesion site as well as the rostral and caudal stumps of the transected spinal cords. The purpose of this study is to examine whether the implanted HUMSCs can induce the regeneration of descending nerve fibres and promote the recovery of hind-limb movement of the spinal cord-injured rats.

## Results

### HUMSC transplantation promotes the regrowth of the injured corticospinal fibers to cross the lesion site

**Figure 1:**
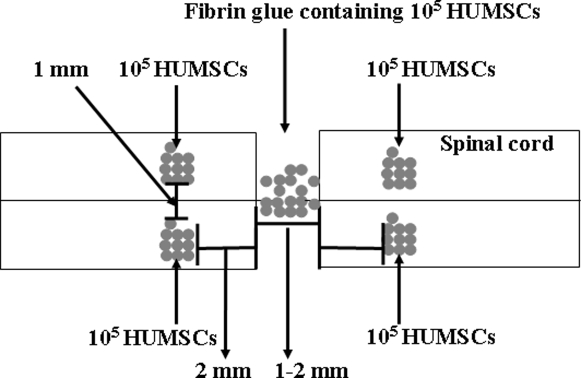
Experimental design for cell grafting after the transection of the spinal cord at the 8th thoracic level.

**Figure 2:**
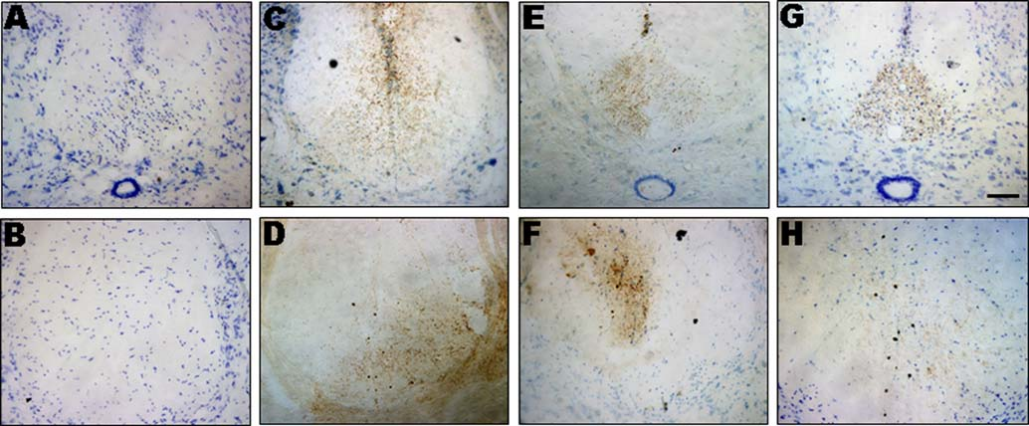
Tracing of the corticospinal tract in the injured spinal cord. Biotinylated dextran amine (BDA) was injected into the bilateral sensorimotor cortex at 16 weeks post-transection. The control group exhibits a few BDA dots in the rostral stump of the injury (A) but not in the caudal stump (B). BDA-positive fibers with brown dots are visible in the rostral and caudal stumps of the lesioned spinal cord in the three transplanted groups (stem cell, NCM-3 and NCM-6 days) (C and D are from the rostral and caudal stumps of the stem cell group; E and F are from the rostral and caudal stumps of the NCM-3 days group; G and H are from the rostral and caudal stumps of the NCM-6 days group). The brown dots indicate the BDA-positive fibers. NCM: neuronal conditioned medium. Scale bar: 100 µm.

Cultured HUMSCs were transplanted into the transected spinal cord (Fig. 1) as described in more details in the Materials and Methods. In order to trace whether the axons in the corticospinal tract had passed the transected lesion site, the tracer biotinylated dextran amine (BDA) was injected into the sensorimotor cortex of the rats 16 weeks after the surgery. The results showed that there were BDA-positive dots in the rostral stump, but not in the caudal stump, of the transected spinal cord in the control group (Fig. 2A, B). This implies that in the control group, the corticospinal fibers had not grown through the transected site. In the three transplanted groups (stem cell, NCM-3, and NCM-6 days groups), both rostral and caudal stumps of the spinal cord had patches of BDA-positive fibers (Fig. 2C–H). These BDA-positive fibers extended up to 1 cm caudal to the implanted site. However, the number of BDA-positive fibers which had extended through the transection to the caudal stumps was significantly lower than the number at the rostral stumps.

### HUMSC transplantation promotes regeneration and provides neuroprotection around the lesion site

**Figure 3:**
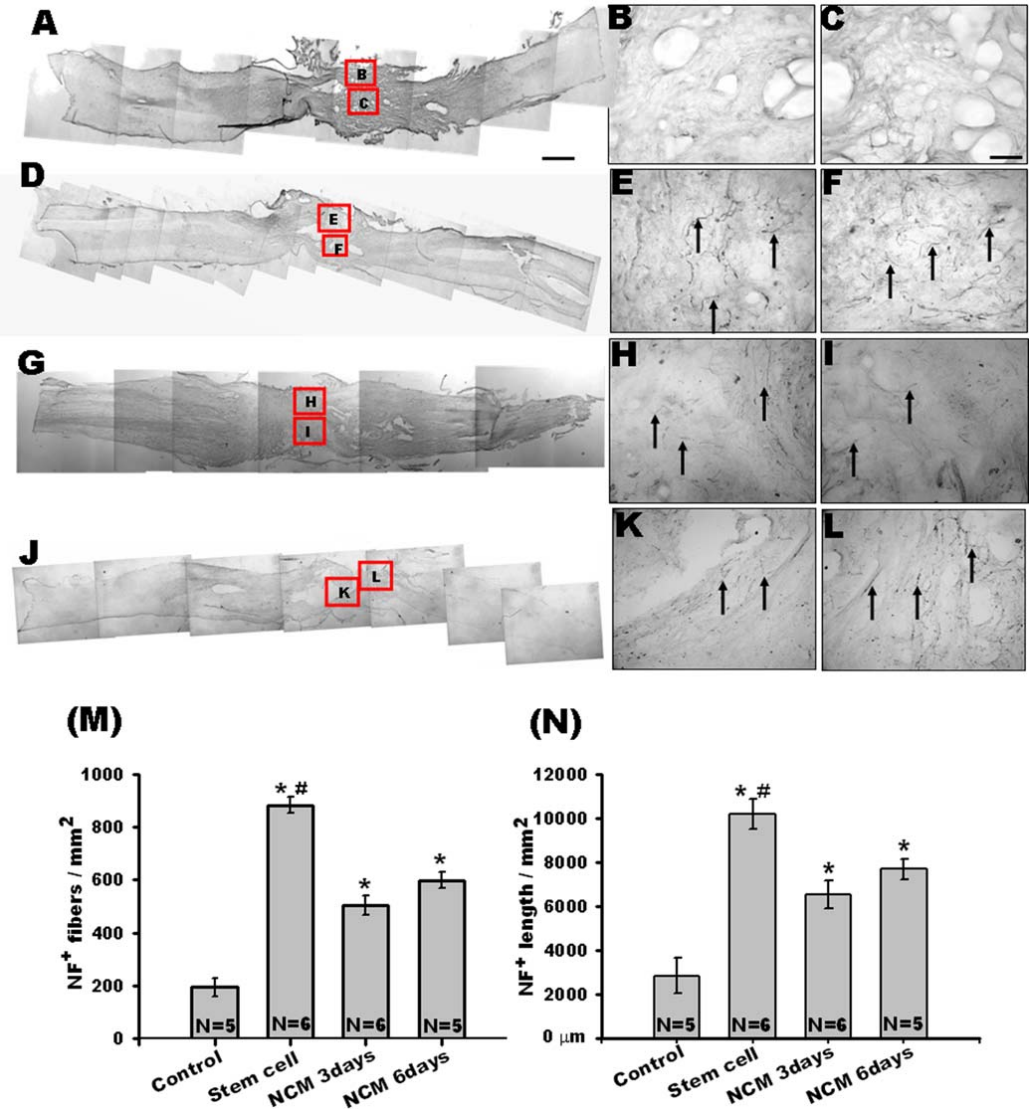
Neurofilament-positive fibers around the lesion 4 months after transection. In A, D, G, and J, the left side is rostral. Representative horizontal sections of the lesion show neurofilament-positive fibers (arrows) in the control group (A–C), stem cell group (D–F), NCM-3 days group (G–I), and NCM-6 days group (J–L). Neurofilament-positive fibers are scarce in the control group. The two right panels are magnified images of the boxed areas. Scale bars: 100 µm in B, C, E, F, H, I, K, L; 1 mm in A, D, G, J. The number (M) and total length (N) of neurofilament-positive (NF^+^) fibers in the transection site of the spinal cord significantly increase in the stem cell group and the groups of NCM-3 days and NCM-6 days, compared with the control group, 4 months after transplantation. * Significant difference at p<0.05 compared with the control group. # Significant difference at p<0.05 compared with the NCM-3 and NCM-6 days groups, respectively.

Sixteen weeks after transection, the neurofilament-positive fibers at the lesion site were scarce in the control group (Fig. 3A–C). Quantification was performed in spinal sections obtained from the transection site. The average number and total length of neurofilament-positive axons in the control group were 193.6±35.3/mm^2^ and 2839.1±802.1 µm/mm^2^ (Fig 3M, N). In the three transplanted groups, the neurofilament-positive fibers at the lesion site were prominently labelled (Fig. 3D–L). In the stem cell group, the average numbers and total length were 882.3±30.1/mm^2^ and 10184.4±669.8 µm/mm^2^, which were statistically greater compared to those of the control group (p<0.05) (Fig. 3M, N). The NCM-3 and NCM-6 days groups displayed significantly higher numbers of axons when compared with the control group (Fig. 3M, N). However, the NCM-3 and NCM-6 days groups displayed significantly lower numbers of axons when compared with the stem cell group (Fig. 3M, N).

### HUMSC grafts change the distribution of astrocytes in spinal cords

**Figure 4:**
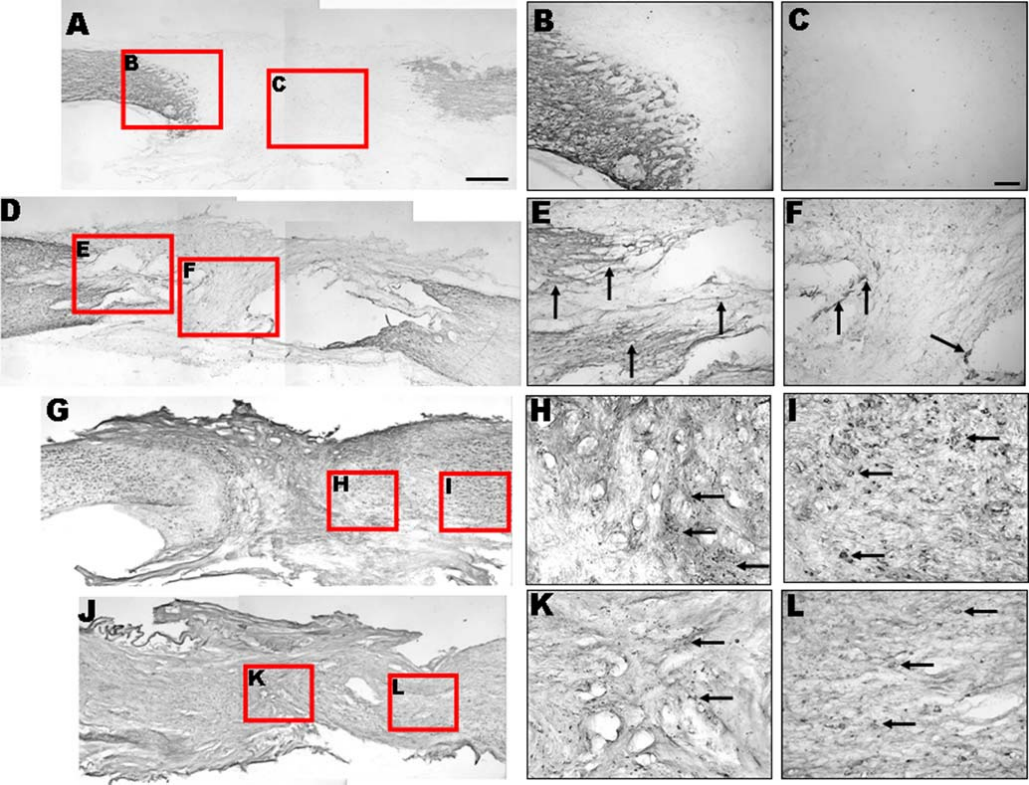
Changes in the distribution of neuroglia in spinal cords after HUMSC transplantation. In A, D, G, and J, the left side is rostral. Representative horizontal sections of the lesion show anti-GFAP (to label astrocytes) (A–F) and anti-ED1 (to label microglia) (G–L) immunostaining 4 months after transection. In the control group, the lesion is bordered by GFAP-positive glial scar (A–C) and in infiltrated by ED1-positive cells (G–I, arrows). In the stem cell group, the glial scar is less dense (D–F, arrows) and ED1-positive microglia are fewer (J–L, arrows) than those in the control group. The two right panels are magnified images of the left boxed areas. Scale bars: 1 mm in A, D, G, J ; 100 µm in B, C, E, F, H, I, K, L.

Anti-glial fibrillary acidic protein (GFAP) immunostaining was performed to assess the distribution of astrocytes 16 weeks after transection. In the control group, astrocytes in the lesion site were scarce (Fig. 4A, C). However, a large number of astrocytes were found at the rostral and caudal stumps. These astrocytes were packed tightly together as a scar barrier (Fig. 4A, B). In the stem cell group, there were a few astrocytes in the lesion site (Fig. 4D, F). There were still many astrocytes at the rostral and caudal stumps, but they appeared to be permissive and did not form a prominent glial limitans to completely block regenerative axons (Fig. 4D, E).

### HUMSC grafts reduce the activation of microglia in spinal cords

Anti-ED1 immunostaining was performed to assess the activation of microglia. In the control group, there were some activated microglia in the lesion site (Fig. 4G, H). Large numbers of activated microglia appeared in the rostral and caudal stumps (Fig. 4G, I). In the stem cell group, there were some activated microglia in the lesion site (Fig. 4J, K). There were fewer activated microglia in the rostral and caudal stumps in the stem cell group (Fig. 4J, L), compared to those of the control group.

### Transplanted HUMSCs survive and migrate in the host spinal cord

**Figure 5:**
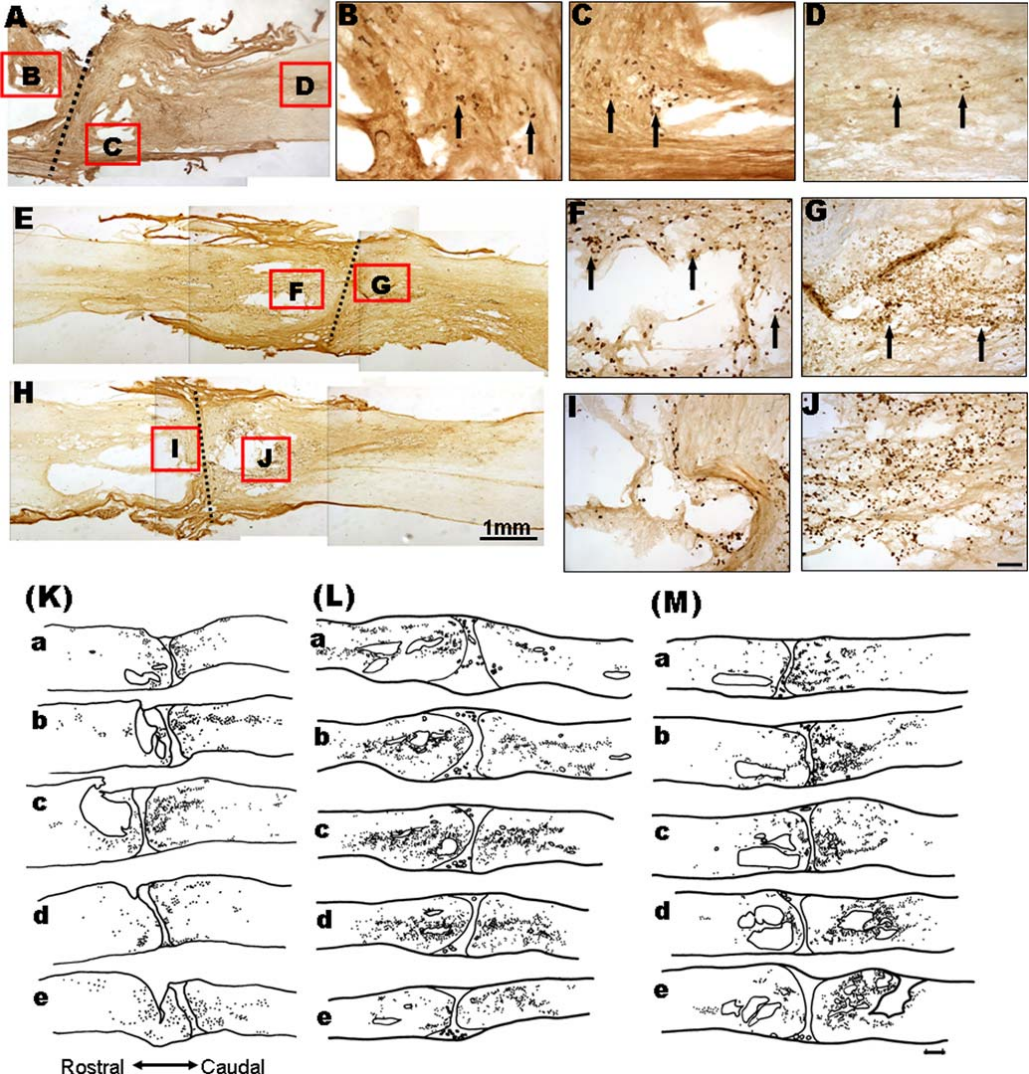
Photomicrographs showing the survival of HUMSCs in rat spinal cords 4 months after transplantation. In A, E, and H, the left side is rostral. Representative horizontal sections show the nuclei of HUMSCs labeled by anti-human specific nuclear antigen in the rostral and caudal stumps of the spinal cord in the stem cell group (A–D), NCM-3 days group (E–G), and NCM-6 days group (H–J). The HUMSCs are microinjected into the transected spinal cords of rats. These cells survive in the spinal cord for 4 months after transplantation. HUMSCs are still evident about 3 mm caudal to the transection in the stem cell group (D). Arrows indicate positively stained cell bodies. The two right panels are magnified images of the boxed areas. The broken line indicates the transection. Scale bars: 1 mm in A, E, H; 100 µm in B–D, F, G, I, J. Line drawings of the rat spinal cord demonstrate the extent of HUMSC migration 16 weeks after implantation in the stem cell group (K), NCM-3 days group (L), and NCM-6 days group (M). In all images, the left side is rostral. Sections a, b, c, d, and e represent every other section of the spinal cord at intervals of 30 µm. •: cells labeled by the anti-human specific nuclear antigen. Scale bar: 500 µm in K–M.

Anti-human specific nuclear antigen immunostaining was used to trace the survival and migration pattern of the HUMSCs. In all three transplanted groups (stem cell, NCM-3, and NCM-6 days groups), HUMSCs survived around the implantation sites at the rostral stump, transection site, and caudal stump (Fig. 5A–J). A series of sections showed that large numbers of HUMSCs survived at least for 4 months after transplantation. The HUMSCs had migrated from the implantation site for about 1.5 mm in the caudal direction of the rostrocaudal axis. The distribution and migration patterns of the implanted HUMSCs in the three transplanted groups did not show apparent differences (Fig. 5K–M).

### Untreated HUMSCs remain undifferentiated in the host spinal cord

**Figure 6:**
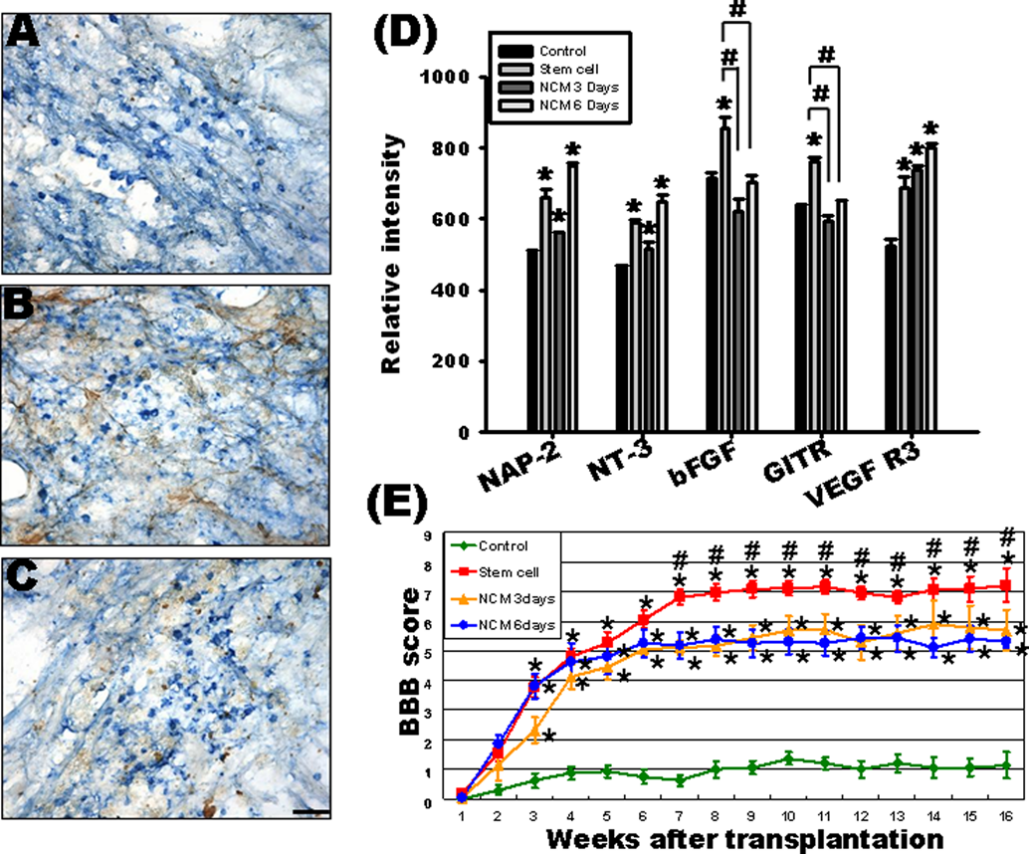
Characterization of HUMSCs in the injured spinal cord and behavioral recovery after HUMSC transplantation. Four months after HUMSC transplantation, the immunostaining of anti-human specific nuclear antigen (blue) in the lesion does not co-localize with that of anti-neurofilament (brown) (A), anti-GFAP (brown) (B), or anti-MBP (brown) (C), indicating that the majority of the implanted HUMSCs have not differentiated into nerve cells, astrocytes, or oligodendrocytes. Scale bar: 50 µm. The relative expression levels of 174 human cytokines are quantified by densitometry (D). The relative intensity of human NAP-2, NT-3, and VEGF R3 are significantly increased in the rat spinal cord of the stem cell, NCM-3, and NCM-6 days groups compared with the control group (* p<0.05). The expression of human bFGF and GITR in the stem cell group are higher than those in the control, NCM-3, and NCM-6 days groups (^#^ p<0.05). (E) The open-field locomotion of rats in different treatment groups is analyzed by the Basso-Beattie-Bresnahan (BBB) Locomotor Rating Scale. The time course of recovery shows treatment-induced benefits start to become apparent at 3 weeks after surgery. The stem cell group shows a statistically significant difference from the NCM-3 and NCM-6 days groups in the seventh week. * indicates a statistical difference between the control group and the three implanted groups (stem cell, NCM-3 and NCM-6 days). # indicates a statistical difference between the stem cell and NCM-3 and NCM-6 days groups. ♦ Control group ▪ Stem cell group ▴ NCM-3 days. • NCM-6 days. (p<0.05 two-way ANOVA followed by LSD test).

Double-staining of human-specific nuclear antigen and neurofilament revealed that most untreated HUMSCs did not differentiate into neurons (Fig. 6A). Staining with anti-human specific nuclear antigen and anti-GFAP showed that the majority of untreated HUMSCs had not differentiated into astrocytes (Fig. 6B). Double-staining of human-specific nuclear antigen and myelin basic protein (MBP) indicated that most untreated HUMSCs were MBP-negative cells, suggesting these cells did not differentiate into oligodendrocytes (Fig. 6C).

### Transplanted HUMSCs express human cytokines and growth factors in the transected rat spinal cord

Spinal cord protein from the rats of control, stem cell, NCM-3 days, NCM-6 days groups were prepared and incubated with membranes containing an array of 174 human protein cytokine antibodies. Autoradiographs were scanned, and the density of each cytokine at the corresponding position was determined. The relative intensities of each cytokine were normalized to control spots on the same membrane. Human neutrophil-activating protein-2 (NAP-2), neurotrophin-3 (NT-3), and vascular endothelial growth factor receptor 3 (VEGF R3) were significantly increased in the rat spinal cord of the stem cell, NCM-3 and NCM-6 days groups (p<0.05) (Fig 6D). The expression of human basic fibroblast growth factor (bFGF) and glucocorticoid induced tumor necrosis factor receptor family **(**GITR) in the stem cell group were higher than those in the other three groups (control, NCM-3 and NCM-6 days) (p<0.05) (Fig 6D).

### HUMSC transplantation improves locomotion recovery

Behavioral testing was performed weekly until 16 weeks post-lesion. No significant improvement was seen during the period of 16 weeks after injury in the control group. Their Basso-Beattie-Bresnahan (BBB) locomotor scores ranged between 0 and 2 points (Fig. 6E, Table 1, and Video S1). Three weeks after transplantation, the first signs of recovery in locomotor function were observed and were statistically significant between the tranplanted groups and the control group. In the stem cell group, BBB scores rose to 6–8 by week 7. This was significantly better than the NCM-3 and NCM-6 days groups (p<0.05, Fig. 6E and Table 1). This trend continued up to 16 weeks. Rats in the stem cell group were able to coordinate movement between the forelimbs and the three joints of the hind-limbs to achieve a walk. Their hind-limbs were also able to lift their bodies off the ground for brief periods, but were unable to support their body weight while walking (Fig. 6E, Table 1, and Video S2).

**Table 1:** BBB scores (means±SE) and animal numbers in each group weekly after spinal cord transection.

	1W	2W	3W	4W	5W	6W	7W	8W
Control	0.00±0.00 (n = 8)	0.31±0.11 (n = 8)	0.63±0.25 (n = 8)	0.88±0.21 (n = 8)	0.94±0.20 (n = 8)	0.75±0.25 (n = 8)	0.63±0.18 (n = 8)	1.00±0.27 (n = 8)
Stem cell	0.19±0.11 (n = 13)	1.58±0.32 (n = 13)	3.77±0.36 (n = 13)	4.81±0.29 (n = 13)	5.27±0.36 (n = 13)	6.04±0.30 (n = 13)	6.81±0.25 (n = 13)	6.96±0.26 (n = 13)
NCM 3 days	0.06±0.06 (n = 9)	1.17±0.53 (n = 9)	2.33±0.43 (n = 9)	4.11±0.41 (n = 9)	4.44±0.41 (n = 9)	5.06±0.21 (n = 9)	5.11±0.23 (n = 9)	5.17±0.33 (n = 9)
NCM 6 days	0.06±0.06 (n = 8)	1.88±0.28 (n = 8)	3.81±0.42 (n = 8)	4.63±0.63 (n = 8)	4.81±0.61 (n = 8)	5.25±0.47 (n = 8)	5.19±0.43 (n = 8)	5.38±0.41 (n = 8)

	9W	10W	11W	12W	13W	14W	15W	16W
Control	1.06±0.22 (n = 8)	1.38±0.21 (n = 8)	1.21±0.21 (n = 7)	1.00±0.29 (n = 7)	1.21±0.31 (n = 7)	1.07±0.35 (n = 7)	1.07±0.30 (n = 7)	1.14±0.45 (n = 7)
Stem cell	7.08±0.29 (n = 13)	7.12±0.23 (n = 13)	7.15±0.23 (n = 13)	6.96±0.23 (n = 12)	6.80±0.21 (n = 10)	7.05±0.38 (n = 10)	6.89±0.43 (n = 10)	7.06±0.57 (n = 10)
NCM 3 days	5.44±0.42 (n = 9)	5.67±0.48 (n = 9)	5.69±0.53 (n = 8)	5.31±0.58 (n = 8)	5.57±0.58 (n = 7)	5.83±0.78 (n = 6)	5.75±0.74 (n = 6)	5.67±0.66 (n = 6)
NCM 6 days	5.25±0.46 (n = 8)	5.31±0.42 (n = 8)	5.25±0.43 (n = 8)	5.44±0.38 (n = 8)	5.43±0.44 (n = 7)	5.17±0.33 (n = 6)	5.50±0.43 (n = 6)	5.42±0.20 (n = 6)

## Discussion

Our study provides evidence that transplantation of human umbilical mesenchymal stem cells from Wharton's jelly is an effective strategy to promote the regeneration of corticospinal fibers and locomotor recovery after spinal cord transection in the rat.

Ideal donor cells for neurological disease therapy should be (i) easily available; (ii) capable of rapid expansion in culture; (iii) immunologically compatible; (iv) capable of long-term survival and integration in the host tissue, and (v) amenable to stable transfection and long-term expression of exogenous genes [12]. HUMSCs in Wharton's jelly of the umbilical cord can be easily obtained and processed, compared to embryonic or bone marrow stem cells. In the present study, approximately 1×10^6^ HUMSCs were collected from a 20 cm-umbilical cord. The number of HUMSCs doubled (2×10^6^) in 10% fetal bovine serum (FBS)-Dulbecco's Modified Eagle Medium (DMEM) in 3 days. In our previous studies, we found that the transformed HUMSCs in the striatum were still viable 4 months after transplantation without the need for immunological suppression [11]. Moreover, transformed HUMSCs survived in the rat liver and were able to control Type 1 Diabetes [13]. Likewise, the HUMSCs in the present study survived in rat spinal cords, suggesting that HUMSCs are an ideal stem cell source for transplantation.

We have demonstrated *in vitro* that approximately 59% of HUMSCs differentiate into neuronal progenitor cells with proliferative ability after 3 days of treatment with NCM, whereas 87% of HUMSCs become immature neurons after 6 days of NCM treatment [9]. Here, the majority of the implanted, untreated HUMSCs in the transected spinal cord remained undifferentiated (Fig. 6A–C). This result contrasts to previous research which demonstrated that embryonic stem cells differentiate into oligodendrocytes [14] or are restricted to a glial lineage [15]. We suggest that the more the surviving stem cells in the host tissue, the higher the possibility for these stem cells to keep undifferentiated and thus to secrete more cytokines and growth factors. As shown in our human cytokine array results, although large amount of human NAP-2, NT-3, and VEGF R3 was secreted in the transected spinal cord of the stem cell (undifferentiated), NCM-3 (differentiated) and NCM-6 (differentiated) days groups, the expressions of human bFGF and GITR in the stem cell group were much higher than those in the other three groups (control, NCM-3 and NCM-6 days) (Fig 6D). Therefore, the mechanism underlying the promotive effect on the regeneration of severed corticospinal axons after the transplantation of HUMSCs is likely the release of more cytokines or growth factors from the undifferentiated stem cells rather than the differentiation of these cells into neuronal or glial cells. Similar conclusions have been reported by Song *et al* [16] and Neuhuber *et al* [17]. They speculate that transplanted bone marrow mesenchymal stem cells facilitate recovery from spinal cord lesions by releasing brain natriuretic peptide and other vasoactive factors that reduce edema, decrease intracranial pressure, and improve cerebral perfusion [17], [18].

NT-3 delivery can improve the growth of corticospinal axons and functional outcomes in chronic stages of injury [19]. Neuronal stem cells constitutively secret NT-3 and promote axonal growth after spinal cord injury [18].

Neovascularization or angiogenesis is necessary for the remodeling of injured tissue. One of the major angiogenic factors is VEGF, which binds to the VEGF Receptor. Signal transduction mediated by this receptor serves various functions in cell proliferation, survival [20], [21], and neovascularization [22], [23]. In response to anoxia, an increase in VEGF concentration and an altered pattern in the expression of VEGF receptor have been found in hippocampal neurons in vitro and in mouse brain, kidney, testis, lung, heart, and liver in vivo [24]–[26]. In addition, VEGF is expressed in cultivated adult neural stem cells isolated from the regions of the rat brain with known spontaneous neurogenesis [27].

Previous research shows that the treatment with bFGF significantly reduces injury zone and protects the survival of motor neurons after spinal cord injury [28]–[30]. However, the underlying mechanisms of bFGF for functional recovery are still not identified yet.

In accordance with the up-regulated cytokines and growth factors, the functional outcomes of the stem cell group are significantly better than those of NCM-3 and NCM-6 day groups in the early stage after spinal cord injury. We speculate that some other cytokines and growth factors beyond the 174 we assessed may also stimulate the regeneration of the injured spinal cord. More interestingly, the cytokines released from transplanted HUMSCs in the injured spinal cord are different from those in the liver with fibrosis (data not shown). We suggest that this heterogeneity in the release of cytokines and growth factors depends on the pathological microenvironments.

Fibrin glue used in this study is a biocompatible tissue adhesive that has been safely applied to the research paradigm of spinal cord injury in rodents and nonhuman primates to facilitate wound healing [31]–[33]. We applied equal amount and concentration of fibrin glue to the transection site in all 4 groups, including the transected control group that received no stem cell transplantation. Therefore, the differences observed among these 4 groups are unlikely a result of fibrin glue usage.

There is increasing evidence that inflammation plays an important role in CNS injury. Experimentally and clinically, CNS damage is followed by an acute and a prolonged inflammatory response characterized by the production of inflammatory cytokines, infiltration of leukocytes and monocytes in the injured area, as well as the activation of resident glial cells, events that may contribute to secondary CNS injury [34]–[36]. Microglial cells are the main effectors of the innate response after CNS injuries. However, whether microglial activation has beneficial or detrimental effects on adjacent damaged neurons remains controversial [37], [38]. There is substantial evidence demonstrating that activated microglia have the potential of releasing cytotoxic factors including nitric oxide, reactive oxygen species, and toxic prostanoids [39], as well as pro-inflammatory cytokines such as tumor necrosis factor-α (TNF-α) or interleukin-1β (IL-1β), which can decrease neuronal functions and promote neurotoxicity [40]–[43]. In addition, attenuation of brain inflammatory response and microglial activation results in neuroprotection in various models of neurodegeneration [44]–[47]. On the other hand, there is increasing evidence suggesting a neuroprotective role for microglia in several pathological conditions of the CNS [48]–[50]. Exogenous application of microglia protects against ischemic injury [51]–[53] and oxygen-glucose deprivation [49], although its underlying molecular mechanisms have so far remained unclear.

After spinal cord injury, astrocytes become hypertrophic, proliferative, and up-regulated in expressing GFAP, and finally form a dense network of glial processes at the lesion site that poses a major physical impediment to regeneration [54]. Astroglial scar formation also inhibits axonal regeneration chemically by producing inhibitory molecules such as chondroitin sulphate proteoglycans [55]–[57]. Degradation of chondroitin sulphate proteoglycans by chondroitinases has been demonstrated to improve functional recovery after brain and spinal cord injuries [58]–[61]. Therefore, it is important to regulate astroglial proliferation and scar formation to create a favorable environment for axonal regeneration.

Our study shows that implanted HUMSCs can modulate the activities of microglia and reactive astrocytes, possibly by releasing certain cytokines, in the injured spinal cord. This is evidenced by the decreased number of microglia and reduced astroglial scarring in the lesion after HUMSCs transplantation (Fig. 4). Similar reports have shown that transplantation of mesechymal stem cells modulates the activation of macrophages or microglia and improves motor function in spinal cord-injured rats [62].

Mesenchymal stem cells have been intensively studied for their potential use in reparative strategies for neurodegenerative diseases and traumatic injuries. Transplantation of mesenchymal stem cells obtained from rat bone marrow significantly improves the recovery of motor function after spinal cord injury [63]. Similar improvements in motor function were found in spinal cord-injured rats receiving human mesenchymal stem cells obtained from adult bone marrow [64]. In our study, the BBB locomotor score of the stem cell group reached the maximum around 7, while the score of the control group consistently remained lower than 2 throughout the entire period of 16 weeks. As demonstrated in our results, such an improvement of motor function after HUMSCs transplantation is associated with abundant neurofilament-positive fibers and regenerative corticospinal axons, reduced microglia infiltration and glial scar formation, and increased expression of cytokines and growth factors around the lesion.

### Conclusion

Our study demonstrates that transplantation of HUMSCs from Wharton's jelly of the umbilical cord is beneficial to wound healing and locomotor recovery after spinal cord injury in rats. Additional research on the functional roles of HUMSCs after transplantation may lead to novel approaches in the future to repairing injured spinal cord.

## Materials and Methods

### Preparation of human umbilical mesenchymal stem cells (HUMSCs)

This experiment was approved by the Research Ethics Committee at the Taipei Veterans General Hospital. With the written consent of the parents, fresh human umbilical cords were obtained after birth and collected in Hanks' Balanced Salt Solution (HBSS) (Gibco, 14185-052, USA) at 4°C. Following disinfection in 75% ethanol for 30 sec, the umbilical cord vessels were cleared off while still in HBSS. The mesenchymal tissue (in Wharton's jelly) was then diced into cubes of about 0.5 cm^3^ and centrifuged at 250 g for 5 min. Following removal of the supernatant fraction, the precipitate (mesenchymal tissue) was washed with serum-free DMEM (Gibco, 12100-046) and centrifuged at 250 g for 5 min. Following aspiration of the supernatant fraction, the precipitate (mesenchymal tissue) was treated with collagenase at 37°C for 18 hours, washed, and further digested with 2.5% trypsin (Gibco 15090-046) at 37°C for 30 min. FBS (Hyclone, SH30071.03, USA) was then added to the mesenchymal tissue to stop trypsinization. The dissociated mesenchymal cells were further dispersed in 10% FBS-DMEM and counted under a microscope with the aid of a hemocytometer. The mesenchymal cells were then used directly for cultures or stored in liquid nitrogen for later use.

### Preparation of neuronal conditioned medium (NCM)

Sprague-Dawley rats at the age of postnatal day 7 were anaesthetized by intraperitoneal injection of overdose pentobarbital (150 mg/kg body weight). The brain was removed, placed in Ca^2+^/Mg^2+^ free buffer (Gibco, 14185-052), and centrifuged at 900 rpm for 5 minutes. Following removal of the supernatant fraction, 10% FBS-DMEM was added to the precipitate (brain tissue). The brain tissue suspension was triturated 15 times for dispersal into single cells. The cells were suspended in 10% FBS-DMEM and incubated at 37°C in 5% CO_2_ and 95% O_2_. In order to inhibit the growth of glial cells, 2 µM AraC (Sigma, c-6645) was added on the next day. On the 5th day of culture, the culture medium, i.e. NCM, was collected to be used for the culture of HUMSCs. The HUMSCs were cultured in NCM alone, which was replaced every other day.

### Animal groups

Female Sprague-Dawley rats (250–300 g body weight) were obtained from the Animal Center of National Yang-Ming University, Taiwan. The Animal Research Committee of the College of Medicine, National Yang-Ming University, approved the study in accordance with the guidelines for the care and use of laboratory animals.

For the different treatments, the rats were divided into four experimental groups: (1) control group, with spinal cord transection and fibrin glue-only in the lesion site (*n* = 8); (2) transection, with HUMSCs and fibrin-glue in the lesion site (referred to as stem cell group; *n* = 13); (3) transection, with HUMSCs treated with NCM for 3 days and fibrin-glue in the lesion site (referred to as NCM-3 days group; *n* = 9); (4) transection, with HUMSCs treated with NCM for 6 days and fibrin-glue in the lesion site (referred to as NCM-6 days group; *n* = 8). NCM-treated or untreated HUMSCs were also implanted in the rostral and caudal stumps of the transected spinal cord in animals receiving HUMSC transplantation.

### Spinal cord transection and HUMSC grafting

Adult female Sprague-Dawley rats were anesthetized with halothane. After a laminectomy at the 7th–9th thoracic vertebral levels, the dura was opened, and the spinal cord was completely transected using a surgical blade. The severed ends of the cord typically retracted about 1–2 mm. The rostral and caudal stumps were lifted to ensure complete transection. Thereafter, 10^5^ HUMSCs were drawn into a glass pipette with a tip diameter of 150–200 µm mounted onto a 5 µl Hamilton syringe (Hamilton, Reno, NV) attached to a micromanipulator. The cells were deposited into two injection sites at the rostral and the caudal stumps, 2 mm from the lesion and 500 µm lateral to the midline, at a depth of 1000 µm. A volume of 5 µl containing 10^5^ HUMSCs in phosphate buffered saline (PBS) was grafted into each site (injection rate: 1 µl/min). Next, 10^5^ HUMSCs in fibrin glue were implanted into the 1–2 mm gap to fill the lesion site in the severed spinal cords. The treatments are illustrated in Fig. 1. After surgery, rats were placed in temperature and humidity controlled incubation chambers until they awoke. They were then transferred to the cages, and bladder evacuation was applied daily. Antibiotics (sodium ampicillin, 80 mg/kg body weight) were injected daily into the rats for a week. The rats were maintained under post-operative care for 16 weeks.

### Assessment of motor function recovery

The Basso-Beattie-Bresnahan Locomotor Rating Scale was used to assess locomotor recovery in an open field [65] by two observers blinded to the animals' identity. Before testing, bladders were expressed, because spontaneous bladder contraction often accompanies hind-limb activity. The rats were placed in an open field and were observed for 5 min. During testing, the activity of animals was also video-monitored.

### The histological study of spinal cords

#### Fixation and sectioning

The rats were perfused with fixative (4% paraformaldehyde and 7.5% picric acid in 0.1 M phosphate buffer (PB) 4 months after surgery. The spinal cord was taken out and immersed in the same fixative at 4°C for 24 hours and then switched to PB containing 30% sucrose before cryosectioning. Successive sections of the spinal cord tissues were sliced at a thickness of 30 µm by using a cryo-microtome and adhered onto gelatin-pretreated slides.

### Immunohistochemistry

Immunohistochemistry was performed by using primary antibodies against 60 kD neurofilament (Chemicon,1∶500), GFAP (to label astrocytes; Chemicon, 1∶1000), ED1 (alternated name CD68 to label microglia; Chemicon, 1∶500) and human specific nuclear antigen (to label cells of human origin; Chemicon, 1∶25), as well as secondary antibodies (biotin-conjugated goat anti-rabbit-IgG, 1∶300 diluted, Sigma and biotin-conjugated goat anti-mouse-IgG, 1∶300 diluted, Sigma), followed by avidin-biotin-horseradish peroxidase complex (ABC KIT, Vector Laboratories, PK-4000) and 3,3′-diaminobenzidine (5 mg in 3.5 µl 30% H_2_O_2_ and 10 ml 50 mM Tris Buffer). Tissue sections were dehydrated and coverslipped with Permount. Pathological changes and quantification of the nerve fibers in the transection site of the spinal cord were observed under an optical microscope and analyzed by *ImagePro* software.

### Assessment of HUMSC differentiation

For the assessment of the possible differentiation of HUMSCs into subpopulations of neurons, astrocytes, or oligodendrocytes, we applied double staining for human-specific nuclear antigen [66] and neurofilament, GFAP, and MBP, respectively.

Spinal cord sections were treated with a blocking solution for 30 min in order to prevent nonspecific antibody-antigen binding. The sections were then reacted with primary antibodies against 60 kD neurofilament (Chemicon, 1∶500), GFAP (Chemicon, 1∶1000), or MBP (Chemicon, 1∶500) at 4°C for 18 hours, washed with 0.1 M PBS, reacted with secondary antibodies (alkaline phosphatase-conjugated goat anti-mouse-IgG for human nuclei, 1∶50; biotin-conjugated goat anti-mouse-IgG, 1∶200, or biotin-conjugated goat anti-rabbit-IgG, 1∶200) at room temperature for 1 hour. After the chromogenic reaction, the sections were coverslipped and observed under a microscope.

### Anterograde tracing of the corticospinal tract

For axon tract tracing, rats received ten stereotaxic injections of 10% biotinylated dextran amine (BDA, MW = 10,000, D-1956, 10% in 0.01 M PBS, Molecular Probes, Eugene) in the sensorimotor cortex 16 weeks after the transection injury. For each injection, 0.4 µl BDA was delivered over a period of 2 min by using a glass pipette. These BDA-injected rats were sacrificed and transcardially perfused with 4% paraformaldehyde (in 0.1 M PB, pH 7.4) 14 days after the injection. The spinal cord and brain were removed and postfixed overnight in the same fixative at 4°C. The spinal cords were cryoprotected in 30% sucrose in PB (0.1 M, pH 7.4) for at least 24 hours, frozen in Tissue-Tek OCT compound (Sakura Finetek, Torrance, CA), and cut into 30-µm-thick sagittal sections. Sections were then processed with avidin-HRP (ABC Elite; Vector Laboratories), followed by a diaminobenzidine HRP reaction for the visualization of the BDA tracer.

### Human protein cytokine array

In order to elucidate which human cytokines were involved in the repair of spinal cord injury, a human protein cytokine kit (RayBio® Human Cytokine Antibody Array C Series 2000, RayBiotech, Inc. AAH-CYT-2000) was used for the human protein cytokine assay 3 weeks after injury. The rat spinal cord, 1.5 cm in length and centered over the lesion, was homogenated and centrifuged in 1× cell lysis buffer at 1,500 *g* to remove cell debris. The membranes included in the human protein cytokine array kit were blocked with a blocking buffer, and then 1 ml of sample supernatant was individually added and incubated at room temperature for 2 h. The membranes were then analyzed according to the manufacturer's instructions.

### Statistical analyses

All data were presented as means±standard error (SE). One-way or two-way ANOVA were used to compare all means, and Least Significant Difference (LSD) was used for the posteriori test. In all statistical analyses p<0.05 was considered significant.

## Supporting Information

Video S1 The locomotion behavior of the control group at week 16 after transection of the spinal cord.
(0.87 MB MPG)

Video S2 The locomotion behavior of the stem cell group at week 16 after transection of the spinal cord.
(6.28 MB MPG)
